# Supplementary material for: Conductometric Studies of Formation the Inclusion Complexes of Phenolic Acids with β-Cyclodextrin and 2-HP-β-Cyclodextrin in Aqueous Solutions
Source: Molecules. 2022 Dec 29;28(1):292. doi: 10.3390/molecules28010292 (PMC9822027; doi:10.3390/molecules28010292)
Supplement: Supplementary file 1 [file molecules-28-00292-s001.zip › molecules-2103519-supplementary.pdf]

Conductometric studies of formation the inclusion complexes of phenolic acids with β-cyclodextrin and 2-HP-β-cyclodextrin in aqueous solutions.

Zdzisław Kinart*

Department of Physical Chemistry, Faculty of Chemistry, University of Lodz, Pomorska 163/165,
90-236 Lodz, Poland
* Correspondence: zdzislaw.kinart@chemia.uni.lodz.pl

Table S1. Densities, viscosities and relative permittivities of pure water at temperatures T = (288.15 to 318.15) K.

T/K	ρ_0 /kg·m^{-3} [a]	$10^3 \cdot \eta$ /Pa·s [a]	ε_r [b]
283.15	999.700	1.130	83.95
288.15	999.100	1.138	82.07
293.15	998.205	1.002	80.21
298.15	997.047	0.890	78.40
303.15	995.651	0.797	76.62
308.15	994.038	0.719	74.89
313.15	992.224	0.653	73.19
318.15	990.223	0.596	71.53

[a] values for water recommended by the International Association for the Properties of Water and Steam (IAPWS) calculated on the online property calculator (https://web1.hszg.de/thermo_fpc/)

[b]values for water calculated according to the IAPWS recommendations (http://www.iapws.org/relguide/dielec.pdf)

Table S2. The value of molar concentration of salt C_{salt} [mol/dm^3], concentration of ligand C_{lig} [mol/dm^3], molar conductivity Λ [S·cm^2·mol^{-1}] for β-cyclodextrin with trans-cinnamic acid sodium salt in water at all tested temperatures at pressure p = 0.1 MPa.[a]

sodium salt of trans-cinnamic acid with β-cyclodextrin									
			283.15	288.15	293.15	298.15	303.15	308.15	313.15
Nr	c_{salt} [mol/dm^3]	c_{lig} [mol/dm^3]	Λ_m [S·cm^2·mol^{-1}]	Λ_m [S·cm^2·mol^{-1}]	Λ_m [S·cm^2·mol^{-1}]	Λ_m [S·cm^2·mol^{-1}]	Λ_m [S·cm^2·mol^{-1}]	Λ_m S·cm^2·mol^{-1}]	Λ_m [S·cm^2·mol^{-1}]
1.	0.00269	0.00000	49.2734	57.8734	69.7734	84.7934	94.9034	106.4034	118.4334
2.	0.00263	0.00030	49.1178	57.7178	69.6178	84.6378	94.7478	106.2478	118.2778
3.	0.00257	0.00060	48.8742	57.4742	69.3742	84.3942	94.5042	106.0042	118.0342
4.	0.00251	0.00091	48.6399	57.2399	69.1399	84.1599	94.2699	105.7699	117.7999
5.	0.00244	0.00121	48.4521	57.0521	68.9521	83.9721	94.0821	105.5821	117.6121
6.	0.00239	0.00149	48.2658	56.8658	68.7658	83.7858	93.8958	105.3958	117.4258
7.	0.00232	0.00181	47.9855	56.5855	68.4855	83.5055	93.6155	105.1155	117.1455
8.	0.00226	0.00208	47.7513	56.3513	68.2513	83.2713	93.3813	104.8813	116.9113
9.	0.00221	0.00235	47.5558	56.1558	68.0558	83.0758	93.1858	104.6858	116.7158
10.	0.00214	0.00270	47.2835	55.8835	67.7835	82.8035	92.9135	104.4135	116.4435
11.	0.00208	0.00299	47.1018	55.7018	67.6018	82.6218	92.7318	104.2318	116.2618
12.	0.00202	0.00329	46.8764	55.4764	67.3764	82.3964	92.5064	104.0064	116.0364
13.	0.00195	0.00359	46.6802	55.2802	67.1802	82.2002	92.3102	103.8102	115.8402
14.	0.00189	0.00388	46.4438	55.0438	66.9438	81.9638	92.0738	103.5738	115.6038
15.	0.00183	0.00416	46.2263	54.8263	66.7263	81.7463	91.8563	103.3563	115.3863
16.	0.00177	0.00448	46.0045	54.6045	66.5045	81.5245	91.6345	103.1345	115.1645
17.	0.00171	0.00477	45.8121	54.4121	66.3121	81.3321	91.4421	102.9421	114.9721
18.	0.00164	0.00506	45.6401	54.2401	66.1401	81.1601	91.2701	102.7701	114.8001
19.	0.00159	0.00534	45.4027	54.0027	65.9027	80.9227	91.0327	102.5327	114.5627
20.	0.00153	0.00564	45.2034	53.8034	65.7034	80.7234	90.8334	102.3334	114.3634
21.	0.00147	0.00591	44.9945	53.5945	65.4945	80.5145	90.6245	102.1245	114.1545
22.	0.00141	0.00620	44.8742	53.4742	65.3742	80.3942	90.5042	102.0042	114.0342
23.	0.00134	0.00654	44.7909	53.3909	65.2909	80.3109	90.4209	101.9209	113.9509

[a] Standard uncertainties are $u(T)$ = 0.01 K, $u(p)$ = 0.05 MPa, $u(c) = 10^{-4}\cdot c$, and the combined expanded uncertainty is $U_c(\Lambda) = 0.0005\cdot\Lambda$ (level of confidence = 0.95).

Table S3. The value of molar concentration of salt C_{salt} [mol/dm^3], concentration of ligand C_{lig} [mol/dm^3],, molar conductivity Λ [S·cm^2·mol^{-1}] for 2-HP-β-cyclodextrin with trans-cinnamic acid sodium salt in water at all tested temperatures..

sodium salt of trans-cinnamic acid with 2-HP-β-cyclodextrin									
			283.15	288.15	293.15	298.15	303.15	308.15	313.15
Nr	c_{salt} [mol/dm^3]	c_{lig} [mol/dm^3]	Λ_m [S·cm^2·mol^{-1}]	Λ_m [S·cm^2·mol^{-1}]	Λ_m [S·cm^2·mol^{-1}]	Λ_m [S·cm^2·mol^{-1}]	Λ_m [S·cm^2·mol^{-1}]	Λ_m S·cm^2·mol^{-1}]	Λ_m [S·cm^2·mol^{-1}]
1.	0.00295	0.00000	45.6472	57.6972	67.2372	82.0872	91.7572	104.4372	114.3672
2.	0.00289	0.00031	45.5263	57.5763	67.1163	81.9663	91.6363	104.3163	114.2463
3.	0.00283	0.00062	45.4610	57.5110	67.0510	81.9010	91.5710	104.2510	114.1810
4.	0.00277	0.00091	45.3736	57.4236	66.9636	81.8136	91.4836	104.1636	114.0936
5.	0.00272	0.00119	45.2867	57.3367	66.8767	81.7267	91.3967	104.0767	114.0067
6.	0.00266	0.00150	45.2067	57.2567	66.7967	81.6467	91.3167	103.9967	113.9267
7.	0.00260	0.00179	45.0917	57.1417	66.6817	81.5317	91.2017	103.8817	113.8117
8.	0.00254	0.00210	45.0073	57.0573	66.5973	81.4473	91.1173	103.7973	113.7273
9.	0.00249	0.00239	44.9153	56.9653	66.5053	81.3553	91.0253	103.7053	113.6353
10.	0.00243	0.00268	44.8407	56.8907	66.4307	81.2807	90.9507	103.6307	113.5607
11.	0.00237	0.00299	44.7526	56.8026	66.3426	81.1926	90.8626	103.5426	113.4726
12.	0.00231	0.00328	44.6649	56.7149	66.2549	81.1049	90.7749	103.4549	113.3849
13.	0.00225	0.00358	44.6168	56.6668	66.2068	81.0568	90.7268	103.4068	113.3368
14.	0.00220	0.00388	44.5703	56.6203	66.1603	81.0103	90.6803	103.3603	113.2903
15.	0.00214	0.00417	44.5152	56.5652	66.1052	80.9552	90.6252	103.3052	113.2352
16.	0.00208	0.00448	44.4494	56.4994	66.0394	80.8894	90.5594	103.2394	113.1694
17.	0.00202	0.00477	44.4083	56.4583	65.9983	80.8483	90.5183	103.1983	113.1283
18.	0.00197	0.00506	44.3633	56.4133	65.9533	80.8033	90.4733	103.1533	113.0833
19.	0.00191	0.00536	44.3356	56.3856	65.9256	80.7756	90.4456	103.1256	113.0556
20.	0.00185	0.00565	44.3038	56.3538	65.8938	80.7438	90.4138	103.0938	113.0238
21.	0.00180	0.00595	44.2657	56.3157	65.8557	80.7057	90.3757	103.0557	112.9857
22.	0.00174	0.00625	44.2456	56.2956	65.8356	80.6856	90.3556	103.0356	112.9656
23.	0.00168	0.00655	44.2042	56.2542	65.7942	80.6442	90.3142	102.9942	112.9242

Table S4. The value of molar concentration of salt C_{salt} [mol/dm^3], concentration of ligand C_{lig} [mol/dm^3],, molar conductivity Λ [S·cm^2·mol^{-1}] for β-cyclodextrin with trans-p-coumaric acid sodium salt in water at all tested temperatures.

			**sodium salt of trans-p-coumaric acid with β-cyclodextrin**						
			283.15	288.15	293.15	298.15	303.15	308.15	313.15
Nr	c_{salt} [mol/dm^3]	c_{lig} [mol/dm^3]	Λ_m [S·cm^2·mol^{-1}]	Λ_m [S·cm^2·mol^{-1}]	Λ_m [S·cm^2·mol^{-1}]	Λ_m [S·cm^2·mol^{-1}]	Λ_m [S·cm^2·mol^{-1}]	Λ_m S·cm^2·mol^{-1}]	Λ_m [S·cm^2·mol^{-1}]
1.	0.002831	0	42.1381	52.4381	59.7581	67.3481	74.7981	83.4481	88.8981
2.	0.002776	0.000293	42.0251	52.3251	59.6451	67.2351	74.6851	83.3351	88.7851
3.	0.002718	0.000603	41.8889	52.1889	59.5089	67.0989	74.5489	83.1989	88.6489
4.	0.002662	0.000903	41.8049	52.1049	59.4249	67.0149	74.4649	83.1149	88.5649
5.	0.002607	0.001199	41.6812	51.9812	59.3012	66.8912	74.3412	82.9912	88.4412
6.	0.002553	0.001489	41.5858	51.8858	59.2058	66.7958	74.2458	82.8958	88.3458
7.	0.002496	0.001794	41.4763	51.7763	59.0963	66.6863	74.1363	82.7863	88.2363
8.	0.002439	0.002103	41.3878	51.6878	59.0078	66.5978	74.0478	82.6978	88.1478
9.	0.002384	0.002394	41.2987	51.5987	58.9187	66.5087	73.9587	82.6087	88.0587
10.	0.00233	0.002688	41.1881	51.4881	58.8081	66.3981	73.8481	82.4981	87.9481
11.	0.002273	0.00299	41.0579	51.3579	58.6779	66.2679	73.7179	82.3679	87.8179
12.	0.002219	0.003282	40.9809	51.2809	58.6009	66.1909	73.6409	82.2909	87.7409
13.	0.002164	0.003579	40.8567	51.1567	58.4767	66.0667	73.5167	82.1667	87.6167
14.	0.002108	0.003878	40.7799	51.0799	58.3999	65.9899	73.4399	82.0899	87.5399
15.	0.002052	0.004177	40.7217	51.0217	58.3417	65.9317	73.3817	82.0317	87.4817
16.	0.001998	0.004466	40.6618	50.9618	58.2818	65.8718	73.3218	81.9718	87.4218
17.	0.001944	0.004758	40.6231	50.9231	58.2431	65.8331	73.2831	81.9331	87.3831
18.	0.001886	0.005068	40.5872	50.8872	58.2072	65.7972	73.2472	81.8972	87.3472
19.	0.001832	0.005357	40.5595	50.8595	58.1795	65.7695	73.2195	81.8695	87.3195
20.	0.001778	0.005649	40.5386	50.8386	58.1586	65.7486	73.1986	81.8486	87.2986
21.	0.001722	0.005948	40.5178	50.8178	58.1378	65.7278	73.1778	81.8278	87.2778
22.	0.001668	0.006238	40.4925	50.7925	58.1125	65.7025	73.1525	81.8025	87.2525
23.	0.001614	0.006528	40.4811	50.7811	58.1011	65.6911	73.1411	81.7911	87.2411

Table S5. The value of molar concentration of salt C_{salt} [mol/dm^3], concentration of ligand C_{lig} [mol/dm^3],, molar conductivity Λ [S·cm^2·mol^{-1}] for 2-HP-β-cyclodextrin with trans-p-coumaric acid sodium salt in water at all tested temperatures.

			sodium salt of trans-p-coumaric acid with 2-HP-β-cyclodextrin						
			283.15	288.15	293.15	298.15	303.15	308.15	313.15
Nr	c_{salt} [mol/dm^3]	c_{lig} [mol/dm^3]	Λ_m [S·cm^2·mol^{-1}]	Λ_m [S·cm^2·mol^{-1}]	Λ_m [S·cm^2·mol^{-1}]	Λ_m [S·cm^2·mol^{-1}]	Λ_m [S·cm^2·mol^{-1}]	Λ_m S·cm^2·mol^{-1}]	Λ_m [S·cm^2·mol^{-1}]
1.	0.002938	0	41.3464	50.6464	57.7964	65.7864	73.9664	82.0564	87.4264
2.	0.00288	0.000299	41.1332	50.4332	57.5832	65.5732	73.7532	81.8432	87.2132
3.	0.002823	0.000597	40.9233	50.2233	57.3733	65.3633	73.5433	81.6333	87.0033
4.	0.002766	0.000891	40.7203	50.0203	57.1703	65.1603	73.3403	81.4303	86.8003
5.	0.002707	0.001194	40.4468	49.7468	56.8968	64.8868	73.0668	81.1568	86.5268
6.	0.002649	0.001492	40.1505	49.4505	56.6005	64.5905	72.7705	80.8605	86.2305
7.	0.002591	0.001791	39.9181	49.2181	56.3681	64.3581	72.5381	80.6281	85.9981
8.	0.002535	0.002084	39.6919	48.9919	56.1419	64.1319	72.3119	80.4019	85.7719
9.	0.002477	0.002382	39.4914	48.7914	55.9414	63.9314	72.1114	80.2014	85.5714
10.	0.002418	0.002685	39.3217	48.6217	55.7717	63.7617	71.9417	80.0317	85.4017
11.	0.00236	0.002985	39.2039	48.5039	55.6539	63.6439	71.8239	79.9139	85.2839
12.	0.002304	0.003276	39.0160	48.3160	55.4660	63.4560	71.6360	79.7260	85.0960
13.	0.002246	0.003573	38.9434	48.2434	55.3934	63.3834	71.5634	79.6534	85.0234
14.	0.002189	0.003868	38.8168	48.1168	55.2668	63.2568	71.4368	79.5268	84.8968
15.	0.002131	0.004166	38.6599	47.9599	55.1099	63.0999	71.2799	79.3699	84.7399
16.	0.002074	0.004464	38.5430	47.8430	54.9930	62.9830	71.1630	79.2530	84.6230
17.	0.002018	0.004754	38.4500	47.7500	54.9000	62.8900	71.0700	79.1600	84.5300
18.	0.001958	0.005061	38.4037	47.7037	54.8537	62.8437	71.0237	79.1137	84.4837
19.	0.001902	0.005348	38.3576	47.6576	54.8076	62.7976	70.9776	79.0676	84.4376
20.	0.001844	0.005648	38.2964	47.5964	54.7464	62.7364	70.9164	79.0064	84.3764
21.	0.001789	0.005933	38.2929	47.5929	54.7429	62.7329	70.9129	79.0029	84.3729
22.	0.001731	0.006234	38.3204	47.6204	54.7704	62.7604	70.9404	79.0304	84.4004
23.	0.001673	0.006535	38.3342	47.6342	54.7842	62.7742	70.9542	79.0442	84.4142

Table S6. The value of molar concentration of salt C_{salt} [mol/dm^3], concentration of ligand C_{lig} [mol/dm^3],, molar conductivity Λ [S·cm^2·mol^{-1}] for β-cyclodextrin with trans-caffeic acid sodium salt in water at all tested temperatures.

sodium salt of trans-caffeic acid with β-cyclodextrin									
			283.15	288.15	293.15	298.15	303.15	308.15	313.15
Nr	c_{salt} [mol/dm^3]	c_{lig} [mol/dm^3]	Λ_m [S·cm^2·mol^{-1}]	Λ_m [S·cm^2·mol^{-1}]	Λ_m [S·cm^2·mol^{-1}]	Λ_m [S·cm^2·mol^{-1}]	Λ_m [S·cm^2·mol^{-1}]	Λ_m S·cm^2·mol^{-1}]	Λ_m [S·cm^2·mol^{-1}]
1.	0.00287	0	36.3781	46.6781	52.9781	59.6281	66.3481	75.4481	81.3581
2.	0.00282	0.00030	36.2651	46.5651	52.8651	59.5151	66.2351	75.3351	81.2451
3.	0.00276	0.00058	36.1289	46.4289	52.7289	59.3789	66.0989	75.1989	81.1089
4.	0.00270	0.00088	36.0449	46.3449	52.6449	59.2949	66.0149	75.1149	81.0249
5.	0.00264	0.00121	35.9212	46.2212	52.5212	59.1712	65.8912	74.9912	80.9012
6.	0.00259	0.00148	35.8258	46.1258	52.4258	59.0758	65.7958	74.8958	80.8058
7.	0.00253	0.00178	35.7163	46.0163	52.3163	58.9663	65.6863	74.7863	80.6963
8.	0.00248	0.00207	35.6278	45.9278	52.2278	58.8778	65.5978	74.6978	80.6078
9.	0.00242	0.00237	35.5387	45.8387	52.1387	58.7887	65.5087	74.6087	80.5187
10.	0.00236	0.00266	35.4281	45.7281	52.0281	58.6781	65.3981	74.4981	80.4081
11.	0.00231	0.00294	35.2979	45.5979	51.8979	58.5479	65.2679	74.3679	80.2779
12.	0.00225	0.00324	35.2209	45.5209	51.8209	58.4709	65.1909	74.2909	80.2009
13.	0.00220	0.00355	35.0967	45.3967	51.6967	58.3467	65.0667	74.1667	80.0767
14.	0.00214	0.00385	35.0199	45.3199	51.6199	58.2699	64.9899	74.0899	79.9999
15.	0.00208	0.00416	34.9617	45.2617	51.5617	58.2117	64.9317	74.0317	79.9417
16.	0.00202	0.00446	34.9018	45.2018	51.5018	58.1518	64.8718	73.9718	79.8818
17.	0.00197	0.00475	34.8631	45.1631	51.4631	58.1131	64.8331	73.9331	79.8431
18.	0.00191	0.00504	34.8272	45.1272	51.4272	58.0772	64.7972	73.8972	79.8072
19.	0.00185	0.00534	34.7995	45.0995	51.3995	58.0495	64.7695	73.8695	79.7795
20.	0.00180	0.00563	34.7786	45.0786	51.3786	58.0286	64.7486	73.8486	79.7586
21.	0.00174	0.00592	34.7578	45.0578	51.3578	58.0078	64.7278	73.8278	79.7378
22.	0.00168	0.00624	34.7325	45.0325	51.3325	57.9825	64.7025	73.8025	79.7125
23.	0.00163	0.00651	34.7211	45.0211	51.3211	57.9711	64.6911	73.7911	79.7011

Table S7. The value of molar concentration of salt C_{salt} [mol/dm^3], concentration of ligand C_{lig} [mol/dm^3],, molar conductivity Λ [S·cm^2·mol^{-1}] for 2-HP-β-cyclodextrin with trans-caffeic acid sodium in water at all tested temperatures.

			**sodium salt of trans-caffeic acid with 2-HP-β-cyclodextrin**						
			283.15	288.15	293.15	298.15	303.15	308.15	313.15
Nr	c_{salt} [mol/dm^3]	c_{lig} [mol/dm^3]	Λ_m [S·cm^2·mol^{-1}]	Λ_m [S·cm^2·mol^{-1}]	Λ_m [S·cm^2·mol^{-1}]	Λ_m [S·cm^2·mol^{-1}]	Λ_m [S·cm^2·mol^{-1}]	Λ_m S·cm^2·mol^{-1}]	Λ_m [S·cm^2·mol^{-1}]
1.	0.00293	0.00000	39.1703	44.3003	50.9703	57.8803	64.9503	73.9203	79.4103
2.	0.00288	0.00027	39.0980	44.2280	50.8980	57.8080	64.8780	73.8480	79.3380
3.	0.00282	0.00058	39.0073	44.1373	50.8073	57.7173	64.7873	73.7573	79.2473
4.	0.00276	0.00089	38.9465	44.0765	50.7465	57.6565	64.7265	73.6965	79.1865
5.	0.00270	0.00118	38.8598	43.9898	50.6598	57.5698	64.6398	73.6098	79.0998
6.	0.00264	0.00148	38.7627	43.8927	50.5627	57.4727	64.5427	73.5127	79.0027
7.	0.00258	0.00177	38.6753	43.8053	50.4753	57.3853	64.4553	73.4253	78.9153
8.	0.00253	0.00206	38.6196	43.7496	50.4196	57.3296	64.3996	73.3696	78.8596
9.	0.00247	0.00236	38.5258	43.6558	50.3258	57.2358	64.3058	73.2758	78.7658
10.	0.00241	0.00268	38.4601	43.5901	50.2601	57.1701	64.2401	73.2101	78.7001
11.	0.00235	0.00297	38.3780	43.5080	50.1780	57.0880	64.1580	73.1280	78.6180
12.	0.00229	0.00326	38.3000	43.4300	50.1000	57.0100	64.0800	73.0500	78.5400
13.	0.00223	0.00356	38.2285	43.3585	50.0285	56.9385	64.0085	72.9785	78.4685
14.	0.00217	0.00385	38.1565	43.2865	49.9565	56.8665	63.9365	72.9065	78.3965
15.	0.00212	0.00415	38.1036	43.2336	49.9036	56.8136	63.8836	72.8536	78.3436
16.	0.00206	0.00444	38.0366	43.1666	49.8366	56.7466	63.8166	72.7866	78.2766
17.	0.00200	0.00474	37.9803	43.1103	49.7803	56.6903	63.7603	72.7303	78.2203
18.	0.00194	0.00504	37.9474	43.0774	49.7474	56.6574	63.7274	72.6974	78.1874
19.	0.00188	0.00532	37.9001	43.0301	49.7001	56.6101	63.6801	72.6501	78.1401
20.	0.00183	0.00562	37.8550	42.9850	49.6550	56.5650	63.6350	72.6050	78.0950
21.	0.00177	0.00590	37.8102	42.9402	49.6102	56.5202	63.5902	72.5602	78.0502
22.	0.00171	0.00621	37.7781	42.9081	49.5781	56.4881	63.5581	72.5281	78.0181
23.	0.00165	0.00650	37.7784	42.9084	49.5784	56.4884	63.5584	72.5284	78.0184

Table S8. The value of molar concentration of salt C_{salt} [mol/dm^3], concentration of ligand C_{lig} [mol/dm^3],, molar conductivity Λ [S·cm^2·mol^{-1}] for β-cyclodextrin with trans-ferulic acid sodium salt in water at all tested temperatures.

sodium salt of trans-ferulic acid with β-cyclodextrin									
			283.15	288.15	293.15	298.15	303.15	308.15	313.15
Nr	c_{salt} [mol/dm^3]	c_{lig} [mol/dm^3]	Λ_m [S·cm^2·mol^{-1}]	Λ_m [S·cm^2·mol^{-1}]	Λ_m [S·cm^2·mol^{-1}]	Λ_m [S·cm^2·mol^{-1}]	Λ_m [S·cm^2·mol^{-1}]	Λ_m S·cm^2·mol^{-1}]	Λ_m [S·cm^2·mol^{-1}]
1.	0.00286	0.00000	38.2905	43.5205	50.8305	55.9705	62.5605	69.8405	77.0565
2.	0.00281	0.00030	38.2204	43.4504	50.7604	55.9004	62.4904	69.7704	76.9864
3.	0.00275	0.00059	38.0883	43.3183	50.6283	55.7683	62.3583	69.6383	76.8543
4.	0.00270	0.00089	37.9823	43.2123	50.5223	55.6623	62.2523	69.5323	76.7483
5.	0.00264	0.00117	37.8822	43.1122	50.4222	55.5622	62.1522	69.4322	76.6482
6.	0.00258	0.00148	37.7979	43.0279	50.3379	55.4779	62.0679	69.3479	76.5639
7.	0.00253	0.00178	37.6908	42.9208	50.2308	55.3708	61.9608	69.2408	76.4568
8.	0.00247	0.00207	37.6202	42.8502	50.1602	55.3002	61.8902	69.1702	76.3862
9.	0.00242	0.00236	37.5575	42.7875	50.0975	55.2375	61.8275	69.1075	76.3235
10.	0.00236	0.00267	37.4855	42.7155	50.0255	55.1655	61.7555	69.0355	76.2515
11.	0.00230	0.00296	37.4225	42.6525	49.9625	55.1025	61.6925	68.9725	76.1885
12.	0.00225	0.00326	37.3625	42.5925	49.9025	55.0425	61.6325	68.9125	76.1285
13.	0.00219	0.00355	37.3006	42.5306	49.8406	54.9806	61.5706	68.8506	76.0666
14.	0.00213	0.00385	37.2442	42.4742	49.7842	54.9242	61.5142	68.7942	76.0102
15.	0.00208	0.00415	37.1702	42.4002	49.7102	54.8502	61.4402	68.7202	75.9362
16.	0.00202	0.00444	37.0894	42.3194	49.6294	54.7694	61.3594	68.6394	75.8554
17.	0.00197	0.00473	37.0316	42.2616	49.5716	54.7116	61.3016	68.5816	75.7976
18.	0.00191	0.00502	36.9555	42.1855	49.4955	54.6355	61.2255	68.5055	75.7215
19.	0.00186	0.00530	36.9059	42.1359	49.4459	54.5859	61.1759	68.4559	75.6719
20.	0.00180	0.00561	36.8825	42.1125	49.4225	54.5625	61.1525	68.4325	75.6485
21.	0.00174	0.00590	36.8462	42.0762	49.3862	54.5262	61.1162	68.3962	75.6122
22.	0.00169	0.00619	36.7849	42.0149	49.3249	54.4649	61.0549	68.3349	75.5509
23.	0.00163	0.00649	36.7664	41.9964	49.3064	54.4464	61.0364	68.3164	75.5324

Table S9. The value of molar concentration of salt C_{salt} [mol/dm^3], concentration of ligand C_{lig} [mol/dm^3],, molar conductivity Λ [S·cm^2·mol^{-1}] for 2-HP-β-cyclodextrin with trans-ferulic acid sodium in water at all tested temperatures.

			**sodium salt of trans-ferulic acid with 2-HP-β-cyclodextrin**						
			283.15	288.15	293.15	298.15	303.15	308.15	313.15
Nr	c_{salt} [mol/dm^3]	c_{lig} [mol/dm^3]	Λ_m [S·cm^2·mol^{-1}]	Λ_m [S·cm^2·mol^{-1}]	Λ_m [S·cm^2·mol^{-1}]	Λ_m [S·cm^2·mol^{-1}]	Λ_m [S·cm^2·mol^{-1}]	Λ_m S·cm^2·mol^{-1}]	Λ_m [S·cm^2·mol^{-1}]
1.	0.00299	0.00000	37.3739	42.4939	49.5139	54.4039	61.2739	68.5939	74.3039
2.	0.00293	0.00031	36.5034	41.6234	48.6434	53.5334	60.4034	67.7234	73.4334
3.	0.00287	0.00060	35.7002	40.8202	47.8402	52.7302	59.6002	66.9202	72.6302
4.	0.00281	0.00090	34.9065	40.0265	47.0465	51.9365	58.8065	66.1265	71.8365
5.	0.00275	0.00118	34.2136	39.3336	46.3536	51.2436	58.1136	65.4336	71.1436
6.	0.00269	0.00149	33.4562	38.5762	45.5962	50.4862	57.3562	64.6762	70.3862
7.	0.00263	0.00180	32.8092	37.9292	44.9492	49.8392	56.7092	64.0292	69.7392
8.	0.00257	0.00208	32.1365	37.2565	44.2765	49.1665	56.0365	63.3565	69.0665
9.	0.00251	0.00240	31.5643	36.6843	43.7043	48.5943	55.4643	62.7843	68.4943
10.	0.00245	0.00269	31.1031	36.2231	43.2431	48.1331	55.0031	62.3231	68.0331
11.	0.00239	0.00298	30.6091	35.7291	42.7491	47.6391	54.5091	61.8291	67.5391
12.	0.00233	0.00328	30.1855	35.3055	42.3255	47.2155	54.0855	61.4055	67.1155
13.	0.00227	0.00358	29.8261	34.9461	41.9661	46.8561	53.7261	61.0461	66.7561
14.	0.00221	0.00388	29.5362	34.6562	41.6762	46.5662	53.4362	60.7562	66.4662
15.	0.00215	0.00417	29.2471	34.3671	41.3871	46.2771	53.1471	60.4671	66.1771
16.	0.00209	0.00447	28.9751	34.0951	41.1151	46.0051	52.8751	60.1951	65.9051
17.	0.00203	0.00476	28.8454	33.9654	40.9854	45.8754	52.7454	60.0654	65.7754
18.	0.00197	0.00506	28.6312	33.7512	40.7712	45.6612	52.5312	59.8512	65.5612
19.	0.00191	0.00536	28.5111	33.6311	40.6511	45.5411	52.4111	59.7311	65.4411
20.	0.00185	0.00566	28.4622	33.5822	40.6022	45.4922	52.3622	59.6822	65.3922
21.	0.00180	0.00594	28.4407	33.5607	40.5807	45.4707	52.3407	59.6607	65.3707
22.	0.00174	0.00624	28.4058	33.5258	40.5458	45.4358	52.3058	59.6258	65.3358
23.	0.00168	0.00653	28.3756	33.4956	40.5156	45.4056	52.2756	59.5956	65.3056
